# Nonbreeder birds at colonies display qualitatively similar seasonal mass change patterns as breeders

**DOI:** 10.1002/ece3.5067

**Published:** 2019-03-29

**Authors:** Louise Emmerson, Sarina Walsh, Colin Southwell

**Affiliations:** ^1^ Australian Antarctic Division Department of Environment and Energy Kingston Tasmania Australia; ^2^ Melbourne Aquarium Melbourne Victoria Australia

**Keywords:** appetite, food requirements, hormonal drivers, *Pygoscelis adeliae*, *Pygoscelis papua*

## Abstract

The difficulty in studying nonbreeding birds means that little is known about them or their resource requirements, despite forming a large and significant component of a population. One way to assess food requirements is to examine changes in body mass, because it indicates the amount of food acquired. In terms of body mass changes, our expectation is that nonbreeders will either (a) be in poorer condition than the breeders which potentially explains why they do not breed or (b) remain at a stable higher mass as they are unconstrained by the physiological costs associated with rearing chicks. Here, we interrogate body mass datasets of breeding and nonbreeding birds of two penguin species to assess these predictions and determine whether differences in mass exist between these two groups throughout the breeding season. The first dataset is from a wild Adélie penguin population, where bird mass was recorded automatically and breeding status determined from a resighting program. A second population of captive gentoo penguins were weighed regularly each breeding season. We demonstrate that although there were times in each year when breeders were heavier than their nonbreeding counterparts for both populations, the mass changes showed qualitatively similar patterns throughout the breeding season irrespective of breeding status. Heavier breeders at times during the breeding season are not unexpected but the overall similar pattern of mass change irrespective of breeding status is in contrast to expectations. It appears that breeding status per se and the constraints that breeding places on birds are not the only driver of changes in mass throughout the breeding season and, although not explicitly studied here, the role of hormones in driving changes in appetite could be key to explain these results. These results present a significant step toward understanding food requirements of nonbreeders in avian populations.

## 1 INTRODUCTION

Nonbreeding birds are difficult to access and study because, unlike breeders which are reliably and regularly accessible while at their colonies or nesting sites during the breeding season, the nonbreeders may or may not return to these sites. For many species of birds, nonbreeders include the younger members of the population which may take several years to return to their breeding colonies (Croxall & Rothery, 1991; Ollason & Dunnet, 1983; Serventy & Curry, 1984; Wooller, Bradley, & Croxall, 1992) and breeding‐aged birds which skip breeding in some years (Goutte, Antoine, Weimerskirch, & Chastel, 2010; Schoech, Mumme, & Moore, 1991; Wynne‐Edwards, 1939). A result of the limited access to nonbreeders is that relatively little is known about them despite forming a significant component of a population. This is unfortunate because the nonbreeders are a crucial “reservoir” of future breeders (Clobert & Lebreton, 1991; Klomp & Furness, 1992; Porter & Coulson, 1987) and our attempts as ecologists to identify, understand, and manage for any threats to a population are thwarted by this fundamental gap in our understanding. This needs to be addressed for effective seabird conservation, to ensure that the resource requirements of total seabird populations are taken into account when setting fisheries catch limits (Southwell et al., 2017; Votier et al., 2008), that threats and impacts distant from breeding colonies are recognized and mediated (e.g., Amélineau et al., 2018), and that monitoring programs are taken into account the lag between an impact on the nonbreeding population and its detection when they recruit into the breeding population (e.g., Jenouvrier, Barbraud, Cazelles, & Weimerskirch, 2005).

Throughout a breeding season, breeding seabirds, the focal group here, are classified as central‐place foragers in that they forage in the waters around their breeding colonies, and return to their nesting sites (or central‐place) to protect and provision their eggs or chicks (Orians & Pearson, 1979). In contrast, the nonbreeders are not necessarily tied to breeding sites, although some can remain associated with the colonies (Ainley, 2002) and some species may benefit from visiting breeding colonies, particularly if that helps maintain or establish territories (e.g., Ewins, 1985; Trivelpiece, Trivelpiece, Geupel, Kjelmyr, & Volkman, 1990; Vleck, Bucher, Reed, & Kristmundsdottir, 1999; Wynne‐Edwards, 1939). During such visits to the colonies, these nonbreeders become accessible for study and this presents an opportunity to extend our understanding about them.

One way of assessing food requirements for seabirds or the impact that changes in the food environment could have on seabird populations is to examine body condition, or changes in body condition or mass, because it indicates the amount of food that an individual has acquired (Fort, Porter, & Grémillet, 2009; Nagy, 1987). There are many measures of body condition, but one of the most reliable and simple is body mass which is a good indicator of fat content (Labocha & Hayes, 2012) and has been used to understand changes in condition for a range of seabirds (Cherel & Freby, 1994; Groscolas et al., 2000; Robin, Boucontet, Chillet, & Groscolas, 1998). During each breeding season, many avian species undergo periods of voluntary fasting which range from a few days to several months (Lewden et al., 2017) and their mass gains and losses fluctuate according to their nest attendance patterns and capacity to ingest food for self‐maintenance (Clarke, Emmerson, & Otahal, 2006; Harris & Wanless, 1988). While a few studies indicate that nonbreeders have a lower mass compared with the breeders at the beginning of the breeding season (Goutte et al., 2010; Trivelpiece et al., 1990; Vleck & Vleck, 2002), such studies typically provide a snapshot in time and little is known about the mass changes of nonbreeders throughout the entire breeding season (Harris & Wanless, 1988).

In addition to differences in body condition, empirical evidence also exists for some seabirds and also terrestrial birds, of a difference in the hormone signature of the breeders compared with the nonbreeders when the decision to breed is made (Goutte et al., 2010; Schoech et al., 1991; Vleck et al., 1999; Vleck & Vleck, 2002). For the younger birds, body condition and hormonal differences are a likely consequence of their immature reproductive systems, social immaturity, less proficient foraging, or inability to attain sufficient condition for breeding (Ainley, 2002; Goutte et al., 2010). The drivers for skipping breeding in breeding‐aged birds are more complex and vary depending on species. For example, it can be a consequence of a natural breeding cycle whereby individuals may breed every second year (Cubaynes, Doherty, Schreiber, & Gimenez, 2011; Goutte et al., 2010; Harris & Wanless, 1988; Wynne‐Edwards, 1939), mistiming between partners (Jeschke, Wanless, Harris, & Kokko, 2007), or once again, under some conditions due to differences in foraging abilities making it difficult for some birds to attain breeding condition (Lescroel et al., 2010; Perrins, 1970; Trivelpiece et al., 1990; Votier et al., 2017). This last point is pertinent because the nature of lengthy nesting duties requires breeding seabirds to forage efficiently when at sea so that they can rapidly gain mass to enable them to sustain mass loss during subsequent periods of fasting when they attend their nests or during molt (Castellini & Rea, 1992; Groscolas & Cherel, 1992).

In the context of the necessity for efficient foraging to attain suitable breeding condition, it is therefore not surprising that breeders are reported to have better body condition at the beginning of the breeding season for some species (Goutte et al., 2010; Trivelpiece et al., 1990; Vleck & Vleck, 2002). However, the expectations for such differences in body condition later in the breeding season are less obvious. For the breeders, body mass changes are dictated by their access to food with some species showing substantial gains and losses related to the length of their nesting duties (e.g., Adélie penguins Ainley, 2002; Emmerson, Clarke, Kerry, & Southwell, 2003; Groscolas & Cherel, 1992; Harris & Wanless, 1988). In contrast, the nonbreeders have no apparent foraging constraints associated with breeding, and hence, if nonbreeders are less efficient foragers, we could expect them to have lower body condition than the breeders throughout the breeding season even if this is indirectly as a consequence of age or experience (Goutte et al., 2010; Harris & Wanless, 1988; Trivelpiece et al., 1990). Equally possible however is the notion that nonbreeders, with a greater capacity to spend time at sea (Prince & Morgan, 1987) and without the energetic costs of incubating eggs or rearing chicks, may have a constant and higher mass than breeders. These expectations, however, are confounded by the notion that there may be an adaptive benefit for not carrying extra weight and that a loss in mass does not necessarily indicate that the bird is under stress (Anker‐Nilssen, Jensen, & Harris, 2018; Groscolas & Robin, 2001; Harris & Wanless, 1988; Myers & Redfern, 2011; Schultner, Kitaysky, Welcker, & Hatch, 2013). In addition, the ability for birds to store energy endogenously is an important mechanism that allows animals to buffer predictable and unpredictable energy requirements (Schultner et al., 2013) which can vary at different stages of their life cycle (Anker‐Nilssen et al., 2018). For the swimming seabirds such as penguins, likely differences in body condition between breeders and nonbreeders are likely to be further exacerbated by physiological limitations of swimming speeds creating a restricted area or “halo” with intense foraging and potential prey depletion around the breeding colonies (Ainley, 2002; Ainley et al., 2004; Birt, Birt, Goulet, Cairns, & Montevecchi, 1987). Because penguins are classic central‐place foragers with potential for prey depletion close to colonies, the energetic costs of breeding are exacerbated, and hence, they are an ideal group of birds to examine predictions relating to seasonal breeder and nonbreeder mass change.

Here, we examine the mass change of two Antarctic‐breeding penguin species across multiple breeding seasons to determine whether differences in mass exist related to the birds’ breeding status (breeders compared with nonbreeders). We do this for a wild population of Adélie penguins *Pygoscelis adeliae* and a captive population of gentoo penguins *Pygoscelis papua*. In this study, the wild Adélie penguin breeders have restricted access to food as a consequence of periods of long fasts while attending their nests (Clarke et al., 2006), whereas the captive gentoo penguins are well fed irrespective of breeding activities. The nonbreeders in both populations are not tied to nests, do not have the energetic constraints of incubating eggs or feeding chicks, and hence have more opportunity to forage. Here, we examine the predictions that (a) nonbreeders have poorer body condition than breeders throughout the breeding season and (b) not having constraints on their foraging activities as a consequence of rearing chicks means that the nonbreeders consistently have higher body condition than the breeders throughout the breeding season.

## 2 METHODS

This study examines the change in penguin mass across multiple breeding seasons for marked individuals from a wild population of Adélie penguins at Béchervaise Island (67°35′S, 62°49′E) off the Mac.Robertson Land coast of East Antarctica and a captive population of the closely related gentoo penguin living in an aquarium in Australia. Data used in this analysis for the Béchervaise Island population include breeding seasons between 1998/99 and 2002/03 and for the gentoo penguins between 2009/10 through until 2014/15. All procedures involving Adélie penguins were approved by the Australian Antarctic Division Animal Ethics Committee, while work on gentoo penguins was with approval of the Melbourne Aquarium Curator and Management.

### 2.1 Species biology and study population description

Adélie and gentoo penguins are closely related species. Adélie penguins have a circumpolar breeding distribution around Antarctica on ice‐free areas on the continent and offshore islands and gentoo penguins breed on sub‐Antarctic islands and the Antarctic Peninsula. Both species breed during the austral summer and build nests made of small pebbles, and moss and tussocks in the case of gentoos.

**Figure 1:**
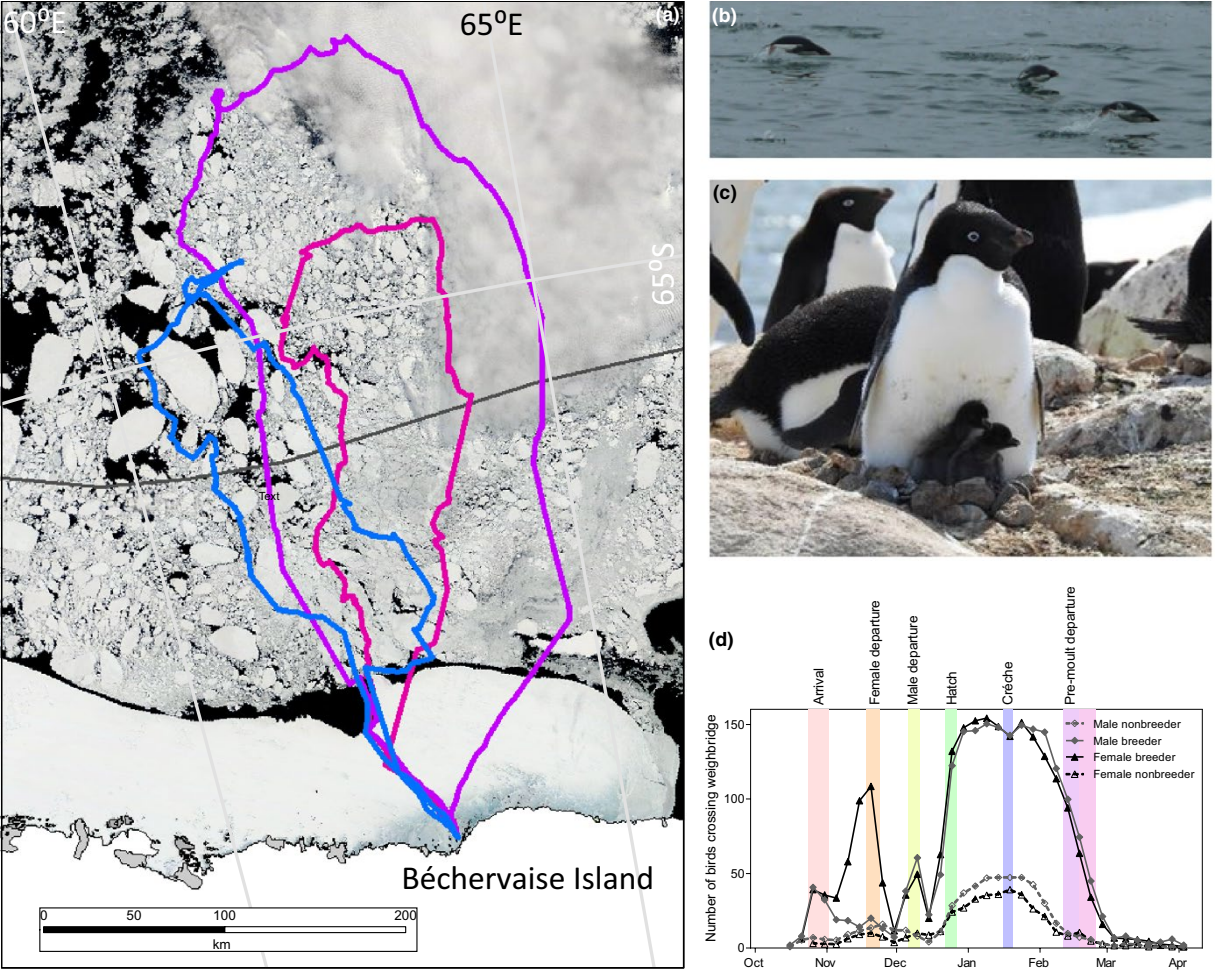
(a) Foraging tracks for three female Adélie penguins during the male incubation period superimposed over satellite imagery (28 November 2014), (b) Adélie penguins porpoising in open water, (c) Adélie penguin protecting chicks at the nest, and (d) the number of Adélie penguin breeder and nonbreeders crossing the weighing platform every 5 days during the breeding season. Colored bands extend between minimum and maximum mean dates for penguin phenology from Emmerson et al. (2011)

The Adélie penguin breeding season commences in October of one year and finishes in late March of the following calendar year (Ainley, 2002). The males arrive first followed by the females several days later, they undergo courtship and build nests, and at the end of November, the female lays two eggs. She departs the colony for a several week long foraging trip which, at some sites, includes a substantial traverse across the fast ice to the open water (Figure 1a,b). After her return, the male forages for a couple of weeks before chicks hatch and enter the “guard” stage where one parent remains with the chick for protection and warmth. At this stage, the small chicks (Figure 1c) require frequent feeds and parent foraging is characterized by short trips and typically loss of mass for the parents (Clarke et al., 2006). Once the chicks are able to thermoregulate and defend themselves (mid‐January), they enter the “crèche” stage where they form groups while both parents forage and can regain body condition. In February, the adults cease feeding the chicks to prepare for molt, and several weeks later, the chicks depart the island to begin their winter migration.

Gentoos exhibit considerable variability in the timing of breeding, and some birds remain at or visit colonies throughout the winter months (Black, Raya Rey, & Hart, 2017). The adults share incubation duties and have short foraging trips with daily changes in nest attendance duties. The chicks start to form crèches at around 26 days and fledge at around 86 days old. Following chick fledging, gentoo adults leave the colony for 1–2 months and later reassemble for molt which takes approximately twenty days (Lynch, 2013). Gentoo penguins require less energy investment when they forage compared with Adélie penguins and therefore do not need as large energy stores as Adélie penguins (D'Amico, Coria, Palacios, Barbosa, & Bertellotti, 2016).

#### 2.1.1 Wild Adélie penguin population

The Béchervaise Island Adélie penguin population is one of 57 Adélie penguin breeding sites along a 70 km stretch of coastline where 445,000 mature (breeding‐age) individual penguins recently bred (Southwell et al., 2017). The island comprises approximately 2,000 nests divided into smaller groups of contiguous nests called subcolonies. At this site, the males arrive around mid‐October followed by the females several days later (Emmerson, Pike, & Southwell, 2011). An automated weighing platform recorded the mass and identity of birds tagged previously with subcutaneously implanted electronic identification devices (Clarke & Kerry, 1998; Kerry, Clarke, & Else, 1993) either as they wandered around the island or visited nests in subcolonies above the gateway. Fences constructed around several of the larger subcolonies direct birds across the gateway. Tagging commenced in 1991/92 with glass coated electronic identification tags (TIRIS, Texas Instruments) measuring 24 or 32 mm long and 3 mm in diameter. These tags are passive transponders which can theoretically last the life time of the bird; details of the technique used for implanting tags and the weighing platform are described in Kerry et al. (1993).

Breeding status was determined from island‐wide resighting of tagged birds on nests and daily nest censuses in study subcolonies. In this study, birds which attempt to breed at the start of the breeding season are termed breeders and nonbreeders are those birds which visit the island but do not attempt to breed. Unfortunately, this study is unable to assess the nonbreeders that do not return to the island at any stage during the breeding season or birds that are not tagged. The term resight here describes the detection of a bird carrying an electronic tag for individual identification and does not involve the physical capture of the bird. The number of breeders and nonbreeders that we recorded crossing the weighing platform was up to 150 (breeders) and 50 (nonbreeders) in any 5‐day period (Figure 1d). These were sufficient for statistical comparisons in most 5‐day periods. Most of the nonbreeder crossings occurred during the period after chicks hatch through until the birds left for preparation for their molt. Fewer nonbreeders crossed the weighbridge early in the breeding season with a small peak at the time when females departed the island for the first foraging trip and again when the males left the island.

Detection of tagged birds on nests during incubation was made across Béchervaise Island from 1998/99 onwards on two occasions each year: (a) 23–29 November, when predominantly breeding males are present at nests and (b) 11–18 December, when predominantly breeding females are present (Kerry et al., 1993). Long‐handled tag readers detected tags at a distance of about 10–20 cm from the bird, which detects in excess of 98% of birds sitting tightly on nests. For the purpose of this paper, all birds detected on nests during these periods are considered to be breeders. Daily nest censuses of adults, eggs, or chicks at nests in several subcolonies were conducted from November when the eggs were laid through until when the chicks crèched and were no longer associated with nests. Birds at nests with eggs were also considered breeders. Nest censuses were conducted from 1991/92 through until 2002/03. For each season, we identified breeding status (breeders or nonbreeders) for known‐sex birds and collated their mass records. For a reduced subset of Adélie penguins which were part of the nest censuses, we assigned breeding status of successful breeders (at least one chick on the nest reached crèche) and failed breeders (those that laid an egg but failed to rear a chick to crèche) to compare with our broader categorization of breeders and nonbreeders across the colony. The period between 1998/99 and 2002/03 presents the best opportunity to accurately determine the breeding status of birds for this study by combining these two datasets, and hence, for this analysis, data are restricted to those years.

#### 2.1.2 Captive gentoo population

**Figure 2:**
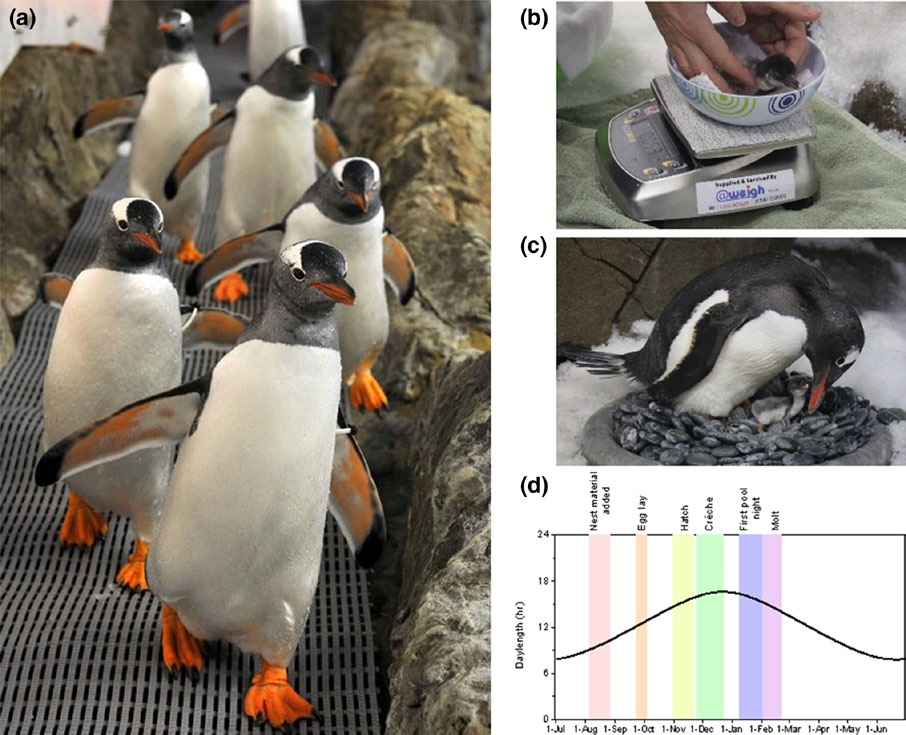
(a) Gentoo penguins walking down the ramp in the enclosure at Melbourne aquarium, Australia, (b) gentoo chick being weighed on scales, (c) parent with chick nesting on cement nest rings and pebbles, and (d) day length hours in enclosure with colored bands showing breeding season phenology

Up to 40 gentoo penguins live in captivity in the Melbourne Aquarium in Victoria, Australia, in an enclosure measuring 80 m^2^ with a pool (surface area 80 m^2^ and volume 120 m^3^) for feeding and swimming (Figure 2a–c). The penguins are hand‐ and pool‐fed daily, and their mass is recorded every few days on scales that the penguins are gently conditioned to step on to. Each penguin is tagged to allow individual identification. The penguins access food through hand‐feeding of whiting and are pool‐fed whitebait, pilchards, squid, and salmon. Feeding continues until the penguins are satiated. Approximately a month prior to breeding, the amount of salmon presented to the penguins increases in response to an increase in demand for feeding from the penguins. In August, each year the keepers place nesting material in the enclosure in the form of nests and pebbles for the penguins to nest with. Nests are monitored daily for eggs, chicks, and the presence of parent adults on nests.

During the breeding season, the penguin mass is recorded, even during incubation shifts. The birds express increased hunger by becoming more insistent during the hand feeds which can include pecking at the keepers’ legs or other birds, some birds become more vocal, honking at the keepers until they are fed in both hand feeds and pool feeds. All birds get access to high fat fish, and nesting birds are fed on the nest so they do not need to leave their nests to search for food. When the amount of food is increased, all birds have access to it; the ones that are not as hungry do not eat as much. The light and temperature profiles in the enclosure remain consistent between years and show a typical annual cycle that matches that expected at sub‐Antarctic penguin colonies (Figure 1d). The breeding season for the captive gentoos commences in September of one year and finishes after they molt in late April of the following calendar year (e.g., 1991/92) aligned with the austral summer.

### 2.2 Data analysis

We analyzed the Adélie and gentoo penguin population mass datasets to assess differences between breeder and nonbreeder mass across and within years. Visual inspection of the data, and previous results from Adélie penguins (Clarke et al., 2006; Emmerson et al., 2003), indicated that the penguins underwent a predictable annual cycle of mass change which was related to the penguins’ breeding phenology and the fasting and feasting associated with incubation, chick rearing, and preparation for molt. There were also differences between years in terms of the absolute value of the peaks and troughs and when they occurred. Here, we performed two assessments of the mass data, the first was to assess differences between breeders and nonbreeders accounting for differences in phenology by choosing key points during the breeding season to compare (across years) and the second was to assess differences between breeders and nonbreeders in every fixed 5‐day period for each year (within years).

To ensure that there was sufficient sample size in each time period for each dataset, we grouped values within 5‐day periods. We removed any birds that were one‐year‐old from analyses as they had not reliably reached adult mass at that age.

#### 2.2.1 Across‐year comparison of mass and date of key mass points

To compare key points in the cycle of annual mass change between years, we interrogated each yearly mass curve to identify peaks, troughs, and transition points when there was an obvious change in the rate of mass gain or loss for each species and sex separately. These were termed key mass points. For the Adélie penguins, there were ten key mass points which occurred at the time of arrival, female predeparture, female departure, male departure, hatch, crèche, fledge, postfledge, premolt peak, and postmolt for the females, and all but the prefemale departure point for the males. In general, these key points occurred when penguins began to prepare for periods of fasting by increasing their mass (peaks at arrival, incubation duty relief (male departure), and premolt Emmerson et al., 2003), the end of periods of mass loss (end of incubation shifts and molt), and in relation to their ability to maintain body condition during the chick‐rearing period (i.e., a general decline when chicks are being guarded and a slow increase during the crèche period, Clarke et al., 2006). There were fewer key mass points for the gentoo penguins which were the same for both sexes and included points when the nest material was added, egg lay, hatch, the first night the chicks spent in the pool, premolt peak, and postmolt. The gentoo penguin key mass points occurred also when penguins began to prepare for egg lay and molt (timing of nest material addition and premolt) and in relation to chick independence.

At each key mass point, we tested for differences between breeders and nonbreeders for both the date of, and the mass for each sex and species using Holm–Sidak multiple *t* tests on yearly means and standard deviations. Significance values were adjusted to account for multiple comparisons. Holm–Sidak multiple *t* tests were conducted with GraphPad Prism version 6 for Windows, GraphPad Software, La Jolla California USA (www.graphpad.com).

#### 2.2.2 Within‐year mass changes

To determine whether mass differed according to breeding status within each year, we examined whether there was a difference between the mass of breeders and nonbreeders during each 5‐day period throughout the breeding season for each sex and species combination. We used linear mixed‐effects models with mass as the response variable, and year (1998/99–2002/03 for Adélie penguins, 2009/10–2014/15 for gentoo penguins), breeding status (breeder or nonbreeder), and 5‐day period (0–72 for gentoo penguins, 22–55 for Adélie penguins) as explanatory variables, and with bird ID as a random factor. Mass data for each sex were analyzed separately for Adélie and gentoo penguins.

Mixed‐effects models were fitted in R v.3.3.3 (R Core Team, 2017) with the “lmer” function in the *lme4* library (Bates & Maechler, 2009; Pinheiro & Bates, 2000) which produces sensible restricted likelihood estimates from unbalanced data which is of benefit in this case. Variance components were estimated with maximum likelihood (ML) to compare models with similar random effects components but different fixed effects structures (Crawley, 2002).

Full statistical models included year, 5‐day period, and status as explanatory variables and all two‐way and three‐way interaction terms. We used a backward selection approach to determine significance of terms with the full model as the starting point. Systematic deletion of each of the fixed effects terms starting with the highest‐order interaction term was examined for their impact on model deviance. The significance of each term after removal was determined from the change in deviance compared against the chi‐square distribution until a minimal model with appropriate random and fixed effects terms was established. Because year was a significant factor for each species and sex, we assessed each year separately starting with the full model including status, 5‐day period, and the interaction term following the same procedure as above. This allowed us to determine whether particular 5‐day periods within a year were more likely to have differences in the mass of breeders and nonbreeders as this was of specific interest. Post hoc comparisons used Tukey's tests using the “lsmeans” function in the *lsmeans* library (Lenth, 2016). P‐values were Bonferroni adjusted to account for multiple comparisons.

## 3 RESULTS

**Figure 3:**
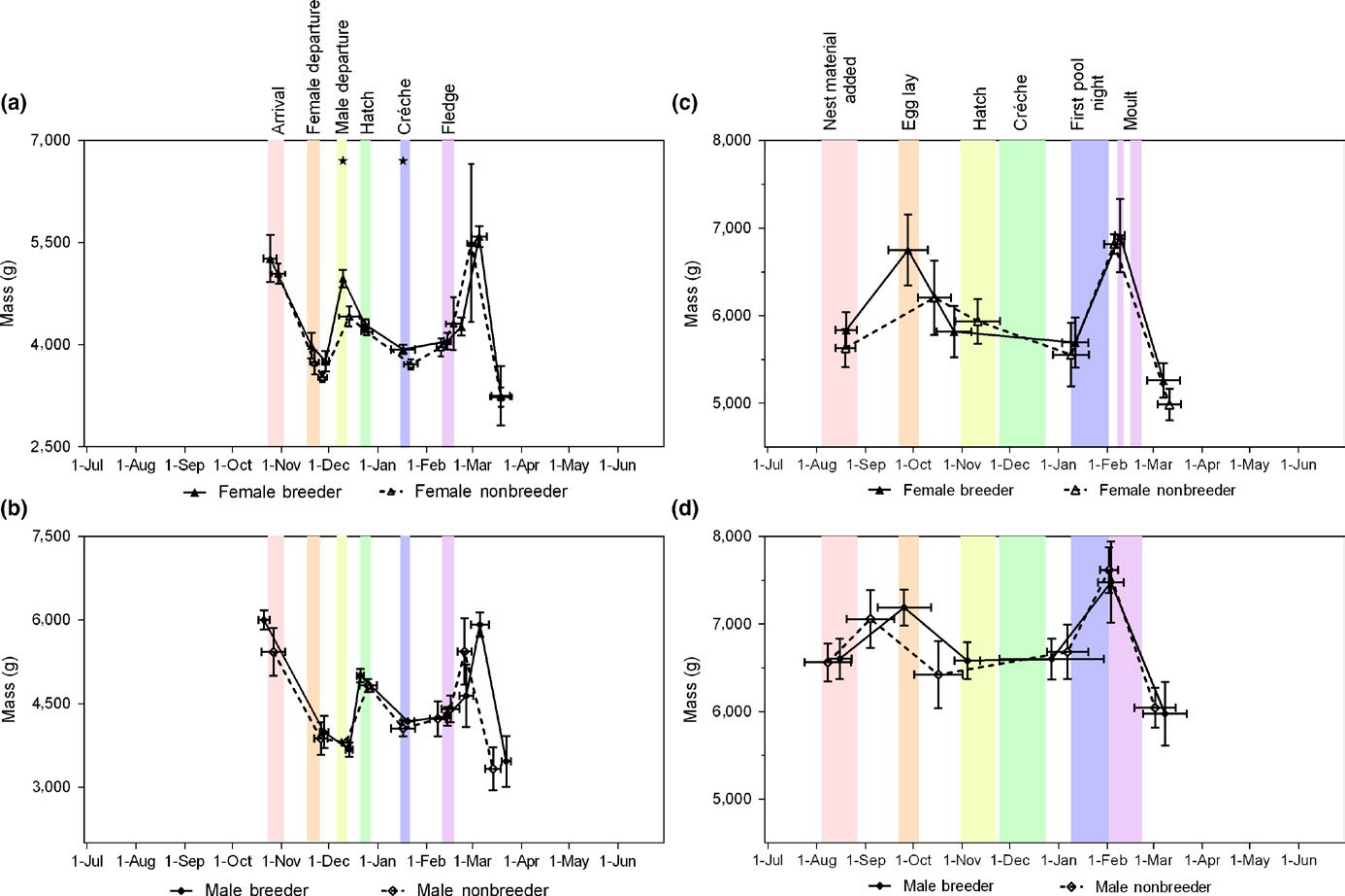
Mean mass at key points during the breeding season for breeder and nonbreeder penguins for (a) female and (b) male Adélie penguins, and (c) female and (d) male gentoo penguins. Values indicate mean and *SD* across years (black lines and symbols). Statistical significance for differences in mass denoted by stars above curves. Colored bands extend between minimum and maximum mean dates for Adélie penguin phenology from Emmerson et al. (2011) relating to key points for mass change

Both male and female Adélie penguins followed a consistent pattern of mass change throughout the breeding season related to the fasting and feasting associated with their nest attendance patterns (Figure 3a: females and b: males; averaged across years). The breeders and nonbreeders for both species showed a similar cycle in mass change patterns. When considering the mass at key points in the annual cycle, the female breeders were heavier than the nonbreeders when they returned from their foraging trip immediately after egg lay (male departure) and also when the chicks crèched (Figure 3a and Table 1). There were no differences in breeder and nonbreeder mass at key points during the breeding season across years for the males or for the dates of key mass points for either male or female Adélie penguins (Figure 3a,b and Table 1 for females and males, respectively).

**Table 1:** Holm–Sidak multiple *t* test comparisons for breeder and nonbreeder mass and dates for Adélie and gentoo penguins. Differences between the mean mass and dates were tested for male and female penguins separately for each key date in the mass change curve

	Males		Females	
	Mass (*p*, *t*‐ratio, *df*)	Dates (*p*, *t*‐ratio, *df*)	Mass (*p*, *t*‐ratio, *df*)	Dates (*p*, *t*‐ratio, *df*)
Adélie penguins				
Arrival	0.03, 2.75, 8	0.14, 1.63, 8	0.22, 1.33, 8	0.1, 1.89, 8
Female predeparture			0.06, 2.14, 8	0.14, 1.63, 8
Female departure	0.54, 0.63, 8	0.4, 0.89, 8	0.02, 3.07, 8	0.24, 1.26, 8
Male departure	0.08, 2.04, 8	0.24, 1.26, 8	*0.0002, 6.3, 8	0.21, 1.37, 8
Hatch	0.05, 2.34, 8	0.1, 1.89, 8	0.03, 2.65, 8	0.61, 0.53, 8
Crèche	0.11, 1.79, 8	0.46, 0.77, 8	*0.002, 4.71, 8	0.24, 1.27, 8
Fledge	0.78, 0.29, 8	0.04, 2.45, 8	0.3, 1.12, 8	0.04, 2.53, 8
Postfledge	0.42, 0.85, 8	0.01, 3.09, 8	0.83, 0.22, 8	0.06, 2.24, 8
Premolt peak	0.24, 1.32, 6	0.04, 2.68, 6	0.88, 0.16, 6	0.11, 1.85, 6
Postmolt	0.71, 0.4, 4	0.07, 2.5, 4	0.96, 0.05, 4	1, 0, 4
Gentoo penguins				
Pre‐increase	0.77, 0.3, 9	0.30, 1.1, 9	0.17, 1.51, 8	0.92, 0.1, 8
First peak	0.44, 0.8, 9	0.06, 2.17, 9	0.08, 2.04, 8	0.06, 2.21, 8
Post first peak	0.41, 0.86, 9	0.03, 2.54, 9	0.53, 0.66, 8	0.09, 1.9, 8
Prehyperphagia	0.80, 0.27, 10	0.70, 0.4, 10	0.49, 0.72, 9	0.66, 0.45, 9
Premolt peak	0.57, 0.59, 9	0.79, 0.27, 9	0.67, 0.45, 8	0.24, 1.26, 8
Postmolt	0.73, 0.36, 9	0.45, 0.78, 9	0.06, 2.24, 8	0.55, 0.62, 8

Significance based on multiplicity adjusted significance levels for multiple comparisons denoted by * and shaded cells.

The captive population of gentoo penguins also had a consistent pattern across years in their change in mass (Figure 3c,d for females and males, respectively) with greater variability between and within years compared with the Adélie penguins. There were no statistically significant differences between the mass and the timing of key points between breeder and nonbreeder gentoo penguins (Figure 3c,d and Table 1).

### 3.1 Within‐year changes in Adélie penguin mass

The three‐way interaction term including year, status, and 5‐day period was significant for male and female Adélie penguin mass data (Table 2). Because our primary interest was in identifying 5‐day periods where differences may exist within years, we proceeded to analyze each year separately. Because the two‐way interaction terms (status and 5‐day period) were significant for Adélie penguins, we conducted Tukey tests to identify where the differences were (Table 2). There was no significant difference in the female breeder and nonbreeder mass at the beginning of the breeding season during arrival and courtship (Supporting Information Figure S1 and Table S1), but in all years, there were 5‐day periods when the nonbreeders weighed less at the time when the females returned to their colonies after their several week foraging trip during incubation (Supporting Information Figure S1). In some years also, the nonbreeders were lighter during some 5‐day periods during the guard, crèche, and premolt peak stages of the breeding season. This difference at the premolt peak may reflect the nonbreeders reaching the peak a little earlier in some years (2000/01 and 2002/03) than the breeders. In contrast to the females, the male breeders were heavier than the nonbreeders in 3/5 years during the arrival and courtship stage (Supporting Information Figure S2 and Table S1). The most likely time of the year for differences between breeder and nonbreeder mass was during late chick rearing and molt preparation. In most years where concurrent breeder and nonbreeder mass was recorded during the premolt period, the nonbreeder males were lighter. The mass change profiles for males and females were qualitatively similar for the breeders and nonbreeders despite some 5‐day periods having a difference between the two.

**Table 2:** Differences in mass for breeder and nonbreeder Adélie and gentoo penguins. Assessment for male and females for each species tested separately with the full model including factors of year, status, and 5‐day period with all two‐way and the three‐way interaction terms. Deletion tests used to examine fixed effects with the change in deviance from the minimal AIC model on removal of a given explanatory variable tested against the chi‐square distribution

	Males	Females
Adélie penguins		
Year * status * 5‐day period	ΔDev = −300, *p < *0.001	ΔDev = −315, *p < *0.001
Two‐way interaction term:		
Year 1998/99	ΔDev = −72, *p < *0.001	ΔDev = −215, *p* < 0.001
Year 1999/2000	ΔDev = −206, *p* < 0.001	ΔDev = −109, *p < *0.001
Year 2000/01	ΔDev = −206, *p* < 0.001	ΔDev = −130, *p* < 0.001
Year 2001/02	ΔDev = −140, *p* < 0.001	ΔDev = −112, *p* < 0.001
Year 2002/03	ΔDev = −85, *p* < 0.001	ΔDev = −19, *p* < 0.001
Gentoo penguins		
Year * status * 5‐day period	ΔDev = −163, *p = *0.0796	ΔDev = −177, *p < *0.001
Year:status	ΔDev = −10.64, *p = *0.06	Not tested
Year:5‐day period	ΔDev = −469, *p* < 0.001	Not tested
Status:5‐day period	ΔDev = −163, *p* < 0.001	Not tested
Year 2009/10		
Two‐way interaction term	ΔDev = −37, *p = *0.026	ΔDev = −23, *p = *0.109
5‐day periods	Not tested	ΔDev = −19.82, *p = *0.228
Status	Not tested	ΔDev = −0.008, *p = *0.928
Year 2010/11		
Two‐way interaction term	ΔDev = −16, *p = *0.860	ΔDev = −41, *p = *0.011
5‐day periods	ΔDev = −186, *p < *0.001	Not tested
Status	ΔDev = −1.46, *p = *0.228	Not tested
Year 2011/12	ΔDev = −105, *p < *0.001	ΔDev = −231, *p < *0.001
Year 2012/13	ΔDev = −77, *p = *0.001	ΔDev = −86, <0.001
Year 2013/14		
Two‐way interaction term	ΔDev = −93, *p < *0.001	ΔDev = −25, *p = *0.489
5‐day periods	Not tested	ΔDev = −265, <0.001
Status	Not tested	ΔDev = −0.225, *p = *0.635
Year 2014/15		
Two‐way interaction term	ΔDev = −16, *p = *0.979	ΔDev = −75, <0.001
5‐day periods	ΔDev = −118, *p < *0.001	Not tested
Status	ΔDev = −0.05, *p = *0.812	Not tested

**Figure 4:**
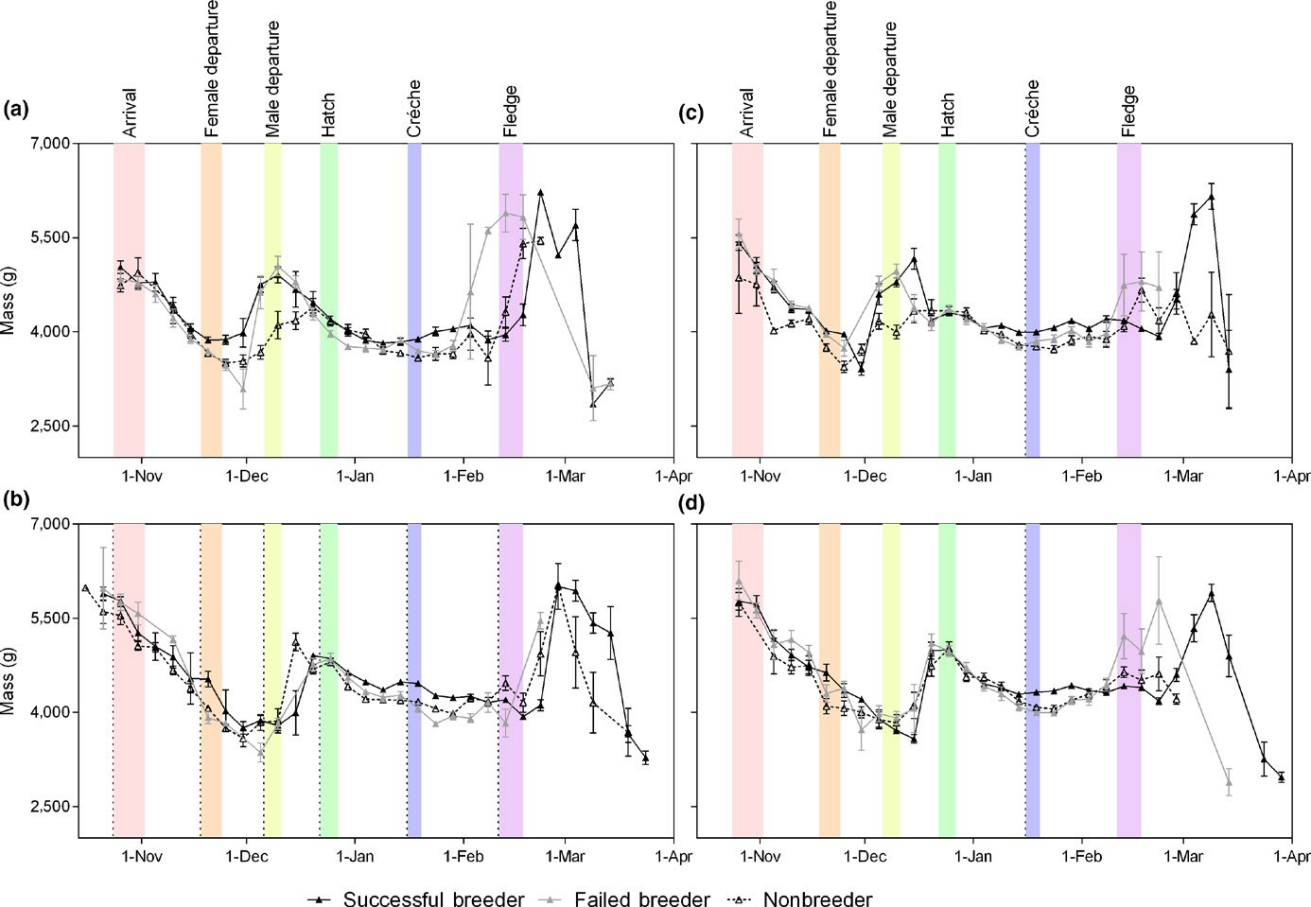
Mean mass every five days for successful, failed, and nonbreeder Adélie penguins for (a) females in 1998 and (b) males in 2001, and (c) females in 2002 and (d) males in 2002. Values are mean and *SEM*. Colored bands extend between minimum and maximum mean dates for Adélie penguin phenology from data presented in Emmerson et al. (2011) relating to key points for mass change

An examination of the difference in mass between successful breeders, failed breeders, and nonbreeders indicated that the failed breeders and the nonbreeders were lighter than the breeders during the late stages of chick rearing (Figure 4). There was little difference in the mass of the failed breeders compared with the breeders prior to the crèche stage. The successful breeders also typically began their premolt hyperphagia a few days later than either the failed breeders or the nonbreeders probably due to their continued need to feed their chicks through until the time when they had fledged.

### 3.2 Within‐year changes in gentoo penguin mass

In the two years with the most pronounced peak in mass associated with egg lay (2011/12 and 2012/13), the nonbreeders had a lower mass at times than the breeders (Supporting Information Table S2). The three‐way interaction term between year, status, and 5‐day period was significant for female gentoo penguins but not for the male mass data (Table 2). However, because terms including year were statistically significant, we proceeded to assess each year's mass change data separately to identify where the differences were. In all cases except 2009/10 for the females, the 5‐day period was highly significant whereas status was only important in conjunction with 5‐day period in several years (2010/11–2012/13). Hence, there was only a difference in mass between breeders and nonbreeders at particular times in some years (Supporting Information Figure S3). There was very little difference toward the end of the breeding season with only two 5‐day periods having a significant difference in mass between the females based on status (2010/11 and 2011/12). The peak in body mass at the beginning of the breeding season was less apparent for the male gentoo penguins (Supporting Information Figure S4). There were no significant differences in body mass between breeder and nonbreeder males until the molt period (Supporting Information Figure S4 and Table S2). During only one 5‐day period, was there a difference between breeder and nonbreeder mass during the nonbreeding period.

## 4 DISCUSSION

In this study, we present a comparison of within‐year mass changes of breeder and nonbreeder birds in a wild Adélie penguin population and a captive gentoo population and in doing so, help fill a substantial gap in our understanding of the nonbreeder mass dynamics. Our results from this multiyear study show two important patterns: Firstly, the consistent annual cyclical changes in body mass throughout the breeding season were similar for both breeding and nonbreeding birds, and secondly, on occasions when there were differences in absolute mass between breeders and nonbreeders within a year, the nonbreeders at the colonies were lighter than the breeders. These results probably reflect several different processes and energy management objectives for the birds depending on the time of year when this was observed in line with similar results reported for other seabird species (Hennin et al., 2016).

For both species, the penguins’ body mass changes were characterized by notable increases immediately prior egg lay and molt, which are two predictably energetically demanding stages of their life cycle (Groscolas & Robin, 2001). The ability to store energy endogenously for periods such as the incubation and molt are well known for many bird species and can be driven by changes in food availability or expected energy expenditure associated with life‐history stages (Schultner et al., 2013). During the chick‐rearing period, penguins’ body mass was lower than that which is physiologically achievable at other times of the breeding season but higher than after their molt and for the Adélie penguins higher than after their initial post/egg lay and incubation fasts (Figure 3). Vleck et al. (1999) suggest that body condition and fat stores of female Adélie penguins may play an important role in determining whether or not a pair breed. For the Adélie penguin population studied here, seasonal reproductive outcomes were previously shown to be related to the mass of the birds departing their colony for their incubation foraging trips but not their mass at arrival (Emmerson et al., 2003). The body mass changes for both species are as expected for a mixed capital‐income breeding strategy with energy storage in preparation for energetically demanding periods (in this case, pre‐incubation) followed by a drop in energy stores during increased mobility periods (in this case, during chick‐rearing activities; Hennin et al., 2016; Schultner et al., 2013). What is less clear is why the nonbreeders had a qualitatively similar pattern in mass change throughout the breeding season despite differences in absolute values on occasion within a year, given that the constraints from breeding were not present.

Based on the expectation that nonbreeders are less able to attain suitable breeding condition (Ainley, 2002; Goutte et al., 2010; Harris & Wanless, 1988; Perrins, 1970; Trivelpiece et al., 1990; Votier et al., 2017), it is intriguing that the differences in mass between breeders and nonbreeders here did not diverge more noticeably, particularly at the start of the breeding season (when the birds arrived at their colonies in the case of the wild Adélie penguin population) or around the time when nesting material was presented (gentoo population). Our predictions that the nonbreeders would either maintain a higher body condition throughout the breeding season as these birds could forage for longer or consistently have lower mass throughout the breeding season as they were less efficient foragers were not supported. Explanations for the similar qualitative pattern of mass change between breeders and nonbreeders therefore need to invoke factors in addition to the opportunity to forage in relation to the constraints of provisioning chicks as drivers of penguin mass throughout the breeding season.

Previous studies have shown differences between female breeder and nonbreeder mass or hormones for a range of bird species at the beginning of a breeding season (Goutte et al., 2010; Groscolas & Robin, 2001; Schoech et al., 1991; Vleck et al., 1999; Vleck & Vleck, 2002) although this is not always the case (Harris & Wanless, 1988). In this study, when we examined the birds mass at key points in the breeding season across years, we found that female Adélie penguin nonbreeders visiting the colony were on average lighter than breeders at the time when the females returned to their nests after their first foraging trip and then again at the start of crèche. Within each year, the main differences occurred after egg lay and during the late stages of chick rearing when successful breeders were heavier than nonbreeders or failed breeders. In particular, the nonbreeders mass did not reach as high a peak immediately prior to chick hatch as the breeders. Whether this was a consequence of breeders being more efficient foragers than nonbreeders, breeders simply having to acquire additional resources to provision chicks, or whether the nonbreeders gain an energetic advantage of being leaner during a more mobile period (Anker‐Nilssen et al., 2018; Hennin et al., 2016; Schultner et al., 2013) is not clear from this study. The pattern is consistent with both the breeders and nonbreeders adapting to their predictable change in allostatic loads throughout the breeding season (Hennin et al., 2016; Schultner et al., 2013). In this study, differences between female breeder and nonbreeder mass within each year occurred more often than differences for the males which is consistent with other empirical studies (Vleck et al., 1999; Vleck & Vleck, 2002).

In light of these results, it is important to consider why seabird mass fluctuates dramatically during the breeding season and what possible mechanisms underlie the similar qualitative pattern reported here for the breeders and nonbreeders for both species and the failed breeders in the case of Adélie penguins. Results from Little Auks (*Alle alle*) suggest that large fat reserves during incubation are beneficial as insurance against being forced to abandon the egg, but after brooding, fat reserves are no longer adaptive and adults may shed surplus fat (Taylor, 1994). Puffins (*Fratercula arctica*) are reported to gain body mass during the winter months as a way of increasing their chance of survival during a period with less predictable food availability and potentially in response to increased energy demands during the colder winter months (Anker‐Nilssen et al., 2018). It is therefore important to acknowledge that changes in mass, even loss, can be an adaptive advantage rather than a constraint for flying and swimming birds (Groscolas & Robin, 2001; Harris & Wanless, 1988; Myers & Redfern, 2011; Taylor, 1994) and that penguins make the “right” reproductive decisions each breeding season based on the integration of available information including their body condition (Vleck & Vleck, 2002). Furthermore, the appetite of birds, especially their re‐feeding appetite after incubation and prior to molt, can be largely triggered by hormones (Angelier et al., 2008; Goymann, Lupi, Kaiya, Cardinale, & Fusani, 2017; Spée et al., 2011; Thierry, Ropert‐Coudert, & Raclot, 2013) which in this case could explain the qualitatively similar pattern of their mass changes if the nonbreeders were subject to similar hormonal triggers to the breeders. Furthermore, hormones, and particularly corticosterone levels, can influence birds foraging decisions, efficiency, food intake, mass gains and losses, and reproductive success (Angelier et al., 2008; Hennin et al., 2016; Thierry et al., 2013). Vleck et al. (1999) claim that the annual and precise cycle in reproductive hormones in both successful and unsuccessful birds is likely regulated by photoperiod but the culmination of the preparation for egg laying may depend heavily on body condition. So important is the hormone cycle that Schoech et al. (1991) state that low levels of sex steroid hormones in nonbreeders may physiologically and behaviorally inhibit reproductive activity so as to render Florida Scrub Jays “reproductively incompetent.” In this context, we suggest that the heavier mass of breeding penguins in some years at some stages of the breeding cycle reported here is probably due to the nonbreeders and possibly the failed breeders being less efficient foragers but that the overall similar cycle of mass change irrespective of breeding status, sex or species reflects a similar hormone cycle experienced by breeders and nonbreeders triggering similar changes in appetite throughout the breeding season. In reality, the role of hormones on foraging behavior may depend also on the nutritional status of the birds (Thierry et al., 2013). For the captive gentoo penguins which are well fed irrespective of breeding status, and with a feeding regime largely driven by the penguins, the consistent annual change in mass and the qualitatively similar pattern between breeders and nonbreeders adds more weight to the role that hormones have on appetite regulation. Hence, the unconstrained nonbreeders may have appetites driven by hormones and behavioral factors from social interactions of being at a colony rather than simply greater opportunities to forage in relation to a lack of constraints imposed by rearing chicks. Unfortunately, concurrent data on hormone levels needed to explore this possibility were not collected as part of this study, and hence, our explanation remains conjecture.

One important consideration for interpreting the results from this study is that the nonbreeder Adélie penguins at Béchervaise Island reported here are those birds that visited the colony at least once during the breeding season. While these birds were classified as nonbreeders if they were not tied to a nest or laid an egg, their presence at the breeding colony, particularly at the start of the breeding season, may indicate that they were intending to breed and may have gone some way toward attaining suitable breeding condition, and so it is perhaps not surprising that some of these birds follow the same cycle as the breeders. In addition, it is also possible that some of the birds classified as nonbreeders may have been very early failed breeders, and while any misclassification of their breeding status is likely to be very low, it is important to keep this in mind. Based on our estimation of the size of the entire nonbreeding population (Southwell et al., 2017), there are also nonbreeders that are part of the total population that remain at sea during the breeding season. Such a large component of pre‐ and nonbreeders is typical for many seabird populations (Jenouvrier et al., 2005). Unfortunately with current approaches, it does not seem possible to measure the mass changes for those Adélie penguins that remain away from the colony during the summer breeding season. Whether those birds follow a similar hormonal cycle that regulates their appetite and their subsequent mass loss and gains is therefore unknown. The fact that the captive gentoo penguins displayed a similar pattern for the breeders and nonbreeders does not shed any light on this as the nonbreeders in that population were subject to social influence and visual cues from the breeders in addition to photoperiod cues and the visual stimuli of the addition of nesting material which could have influenced their behavior and hence appetite. Very few studies have been able to access birds away from their colonies, and one that has, has relied upon birds being caught at sea in an annual winter harvest (Anker‐Nilssen et al., 2018). While that study focussed on the nonbreeding winter period, the results suggest that changes in body mass throughout the birds' life cycle can have demographic advantages and be responsive to the predictability of the environment and that an understanding of the energy requirements based on environmental conditions and the birds' allostatic load during the entire life cycle is important for understanding the context of body mass changes.

The results from our long‐term mark‐resight and monitoring program, and an automated weighing platform have allowed us to understand mass change patterns of Adélie penguins in relation to breeding status and hints strongly at the role that hormones play in regulating their appetite to sustain such mass changes. Data from the weighing platform fill a substantial data gap on the annual cycle of mass changes in Adélie penguins. Adélie penguins are central‐place foragers during the breeding season and can have substantial distances to traverse across the ice to reach their foraging grounds (Emmerson & Southwell, 2008). Their well‐recognized fasting and feasting cycle throughout the breeding season (Ainley, 2002) can be exacerbated by the extent of the fast ice they encounter on their way to forage which is dynamic within a breeding season and highly variable across breeding seasons (Emmerson et al., 2011; Emmerson & Southwell, 2008). Comparing the mass change patterns of breeders and nonbreeders for both a wild Adélie penguin population as well as a captive gentoo population which was well fed irrespective of nesting and chick provisioning duties has enabled us to develop a greater appreciation of the drivers of seasonal penguin mass changes for breeding and nonbreeding penguins. To our knowledge, this is the first study to do this for both breeders and nonbreeders throughout an entire breeding season.

In conclusion, the results from this study on sex‐ and species‐specific changes in body mass in relation to breeding status during a breeding season present a significant step toward estimating the annual cycle of resource and food requirements for the important nonbreeder component of a seabird population. In future studies, it would be worthwhile confirming the role that hormones play in relation to body condition and appetite for both wild populations as well as their captive counterparts (Spée et al., 2011). Attempts to obtain data on the currently unstudied nonbreeders that remain at sea are crucial and will require creative research approaches. While our focus here is on the nonbreeders that visit the Adélie penguin colony rather than those birds that remain at sea throughout the breeding season and could have a different mass change pattern, our study presents a substantial contribution toward understanding the requirements of the nonbreeder population. Results from studies like this can be used to formulate specific hypotheses for the regulation of food intake, fat storage, decisions to abandon the nest, and the subsequent reproductive outcomes in an environment that is earmarked for great change in relation to food availability and quality in the future (Constable et al., 2014).

## CONFLICT OF INTEREST

None declared.

## AUTHORS CONTRIBUTIONS

LE and CS conceived the ideas, designed the methodology, and collated the Adélie penguin data. LE analyzed the data. SW collected and collated the gentoo penguin data. All authors contributed critically to the writing of the manuscript and gave final approval for publication.

## Data Availability

Data will be archived through the Australian Antarctic Division Data Centre (AADC) and will be available upon request to the data curators in line with the data management plan associated with Australian Antarctic Science project AAS #4087 subject to a 12‐month embargo period after manuscript publication.
