# Supplementary material for: Nonbreeder birds at colonies display qualitatively similar seasonal mass change patterns as breeders
Source: Ecol Evol. 2019 Mar 29;9(8):4637–50. doi: 10.1002/ece3.5067 (PMC6476789; doi:10.1002/ece3.5067)
Supplement: Supplementary file 1 [file ECE3-9-4637-s001.docx]

## Table S1:

|  | | **Males** | | | | | **Females** | | | | |
| --- | --- | --- | --- | --- | --- | --- | --- | --- | --- | --- | --- |
|  | 5-day period | 1998/99 | 1999/2000 | 2000/01 | 2001/02 | 2002/03 | 1998/99 | 1999/2000 | 2000/01 | 2001/02 | 2002/03 |
| arrival and courtship | 22 |  |  |  | 0.032 |  |  |  |  |  |  |
|  | 23 | 0.061 |  |  | 0.071 | 0.579 | 0.178 |  |  | 0.003 |  |
|  | 24 | 0.160 | <0.001 |  | <0.001 |  | 0.916 | 0.819 | 0.950 | 0.210 | 0.621 |
|  | 25 | 0.496 |  | 0.307 | 0.371 | 0.082 |  | 0.437 | 0.333 | 0.062 | 0.104 |
|  | 26 | 0.133 | 0.881 | 0.180 | 0.002 | 0.163 | 0.875 | 0.177 | 0.053 | 0.009 | 0.158 |
|  | 27 | <0.001 | 0.080 | 0.171 | 0.457 | 0.297 | 0.744 | 0.122 | 0.100 | 0.009 | 0.259 |
| incubation | 28 | 0.075 | 0.005 | 0.003 | 0.016 | 0.009 | 0.055 | 0.006 | 0.220 | <0.001 | 0.015 |
|  | 29 | 0.064 | 0.003 | 0.137 | 0.030 | 0.269 | 0.016 | 0.089 | 0.436 | 0.004 | 0.024 |
|  | 30 | 0.430 | 0.635 | 0.717 | 0.169 | 0.874 | 0.083 | 0.037 | 0.242 | <0.001 | 0.342 |
|  | 31 | 0.551 | 0.218 | 0.518 | 0.576 | 0.641 | <0.001 | <0.001 |  | <0.001 | <0.001 |
|  | 32 | 0.576 | 0.834 | 0.428 | 0.699 | 0.759 | <0.001 | 0.025 | <0.001 | <0.001 | <0.001 |
|  | 33 | 0.093 | 0.694 | 0.228 | 0.011 | 0.032 | <0.001 | 0.025 | 0.003 | 0.589 | 0.109 |
|  | 34 | 0.645 | 0.010 | 0.013 | 0.098 | 0.212 | 0.896 | 0.004 | 0.891 | 0.942 | 0.216 |
| guard | 35 |  | 0.106 | 0.382 | 0.979 | 0.867 |  | 0.883 | 0.234 | 0.067 | 0.109 |
|  | 36 | 0.079 | 0.842 | 0.577 | <0.001 | 0.366 | 0.111 | 0.298 | 0.002 | 0.013 | 0.090 |
|  | 37 | 0.001 | 0.028 | 0.404 | <0.001 | 0.131 | 0.213 | 0.109 | 0.005 | <0.001 | 0.565 |
|  | 38 | 0.061 | 0.595 | 0.105 | 0.018 | 0.615 | 0.015 | 0.186 | 0.027 | <0.001 | 0.998 |
| crèche | 39 | 0.101 | 0.605 | 0.036 | <0.001 | 0.228 | 0.301 | 0.037 | 0.613 | <0.001 | 0.086 |
|  | 40 | <0.001 | 0.657 | 0.001 | <0.001 | 0.004 | 0.053 | 0.048 | 0.002 | <0.001 | 0.010 |
|  | 41 | <0.001 | 0.041 | 0.001 | 0.001 | 0.002 | 0.003 | 0.007 | 0.007 | <0.001 | <0.001 |
|  | 42 | <0.001 | 0.050 | <0.001 | 0.002 | 0.060 | <0.001 | 0.014 | <0.001 | 0.028 | <0.001 |
|  | 43 | <0.001 | <0.001 | 0.126 | 0.621 | 0.093 | <0.001 | 0.062 | 0.112 | <0.001 | 0.103 |
|  | 44 | 0.167 | 0.038 | 0.014 | 0.537 | 0.365 | 0.681 | 0.719 | 0.995 | 0.110 | 0.012 |
| fledge and moult | 45 | 0.476 | 0.392 | 0.180 | 0.007 | 0.576 | 0.435 | 0.105 | 0.289 | 0.893 | 0.155 |
|  | 46 | 0.004 | 0.497 | <0.001 | 0.409 | 0.331 | 0.650 |  | 0.030 | 0.481 | <0.001 |
|  | 47 | 0.522 | <0.001 | 0.866 | <0.001 | 0.018 | <0.001 |  | 0.039 | 0.317 | 0.752 |
|  | 48 |  | <0.001 | 0.337 | 0.669 | 0.528 | 0.943 |  | <0.001 | 0.073 | 0.665 |
|  | 49 |  |  | <0.001 | 0.013 | 0.049 |  |  | <0.001 |  | <0.001 |
|  | 50 |  |  | <0.001 | <0.001 |  |  |  | 0.743 |  | 0.464 |
|  | 51 |  |  | 0.055 | 0.002 | 0.033 |  |  | <0.001 |  | 0.845 |
|  | 52 |  |  |  |  |  |  |  | 0.005 |  |  |
|  | 53 |  |  |  |  |  |  |  |  |  |  |
|  | 54 |  |  | 0.416 |  |  |  |  | 0.024 |  |  |
|  | 55 |  |  |  |  |  |  |  |  |  |  |
| Bonferroni  P-value | | 0.0021 | 0.0020 | 0.0017 | 0.0019 | 0.0017 | 0.0019 | 0.0019 | 0.0017 | 0.0016 | 0.0019 |

Table S1: Tukey test results from comparisons between breeders and non-breeder Adélie penguins in each year. Tukey test results conducted when two-way interactions were significant in backward selection procedure for each year and sex in Table 2. Because of multiple comparisons, the P-values were Bonferroni adjusted based on the number of comparisons being conducted. 5-day period 22 covers the period from 19-23 October.

## Table S2:

|  | | **MALE** | | | | | | **FEMALE** | | | | | |
| --- | --- | --- | --- | --- | --- | --- | --- | --- | --- | --- | --- | --- | --- |
|  | 5-day periods | 2009/10 | 2010/11 | 2011/12 | 2012/13 | 2013/14 | 2014/15 | 2009/10 | 2010/11 | 2011/12 | 2012/13 | 2013/14 | 2014/15 |
| non-breeding period | 0 | 0.794 | Status not significant – not tested |  | 0.462 | 0.855 | Status not significant – not tested | Status not significant – not tested |  |  | 0.442 | Status not significant – not tested | 0.990 |
|  | 1 | 0.698 |  | 0.858 |  |  |  |  |  | 0.281 |  |  | 0.988 |
|  | 2 | 0.893 |  | 0.342 | 0.545 | 0.528 |  |  |  | 0.145 | 0.726 |  |  |
|  | 3 |  |  |  | 0.903 | 0.664 |  |  |  |  | 0.497 |  | 0.906 |
|  | 4 |  |  | 0.740 | 0.382 | 0.672 |  |  |  | 0.073 | 0.351 |  | 0.993 |
|  | 5 | 0.627 |  |  |  |  |  |  |  |  |  |  |  |
|  | 6 |  |  | 0.696 |  | 0.907 |  |  |  | 0.069 |  |  | 0.881 |
|  | 7 | 0.760 |  |  |  | 0.704 |  |  |  |  |  |  | 0.902 |
|  | 8 | 0.940 |  | 0.896 | 0.525 |  |  |  |  | 0.022 | 0.027 |  | 0.740 |
| courtship | 9 | 0.691 |  | 0.917 | 0.376 | 0.701 |  |  |  | 0.036 | 0.050 |  |  |
|  | 10 |  |  |  | 0.484 | 0.280 |  |  |  |  | 0.017 |  | 0.729 |
|  | 11 | 0.888 |  | 0.705 |  | 0.326 |  |  |  | 0.004 |  |  |  |
|  | 12 | 0.526 |  | 0.667 | 0.442 |  |  |  |  | 0.003 | 0.103 |  |  |
|  | 13 |  |  | 0.942 | 0.006 | 0.070 |  |  |  | 0.003 | 0.120 |  | 0.157 |
| incubation | 14 | 0.560 |  |  | 0.016 | 0.101 |  |  |  |  | 0.024 |  | 0.109 |
|  | 15 | 0.445 |  | 0.662 |  |  |  |  |  | 0.001 |  |  | 0.667 |
|  | 16 | 0.904 |  | 0.507 | 0.119 | 0.015 |  |  |  | <0.001 | 0.015 |  |  |
|  | 17 |  |  |  |  | 0.025 |  |  |  |  | 0.002 |  | 0.797 |
|  | 18 | 0.252 |  | 0.814 |  | 0.027 |  |  |  | <0.001 |  |  | 0.097 |
|  | 19 | 0.608 |  | 0.604 |  |  |  |  |  | <0.001 | 0.001 |  |  |
|  | 20 |  |  | 0.586 | 0.115 | 0.007 |  |  |  | <0.001 | 0.514 |  |  |
|  | 21 |  |  |  |  | 0.022 |  |  |  |  | 0.399 |  | 0.002 |
|  | 22 | 0.316 |  | 0.948 |  |  |  |  |  | 0.076 |  |  | <0.001 |
|  | 23 |  |  | 0.696 | 0.410 |  |  |  |  |  | 0.229 |  |  |
|  | 24 | 0.386 |  |  | 0.114 | 0.178 |  |  |  |  |  |  |  |
| Guard | 25 | 0.384 |  | 0.934 |  |  |  |  |  | 0.025 |  |  |  |
|  | 26 | 0.924 |  |  | 0.082 |  |  |  |  |  | 0.074 |  |  |
|  | 27 |  |  | 0.743 | 0.007 |  |  |  |  | 0.430 |  |  | 0.156 |
|  | 28 | 0.890 |  |  |  |  |  |  |  |  |  |  | 0.019 |
|  | 29 |  |  | 0.331 |  |  |  |  |  | 0.301 |  |  | 0.051 |
|  | 30 |  |  | 0.838 | 0.518 | 0.474 |  |  |  | 0.506 | 0.679 |  |  |
|  | 31 | 0.633 |  | 0.726 |  | 0.445 |  |  |  | 0.897 | 0.814 |  | 0.101 |
| crèche | 32 | 0.427 |  |  |  | 0.650 |  |  |  |  |  |  | 0.225 |
|  | 33 | 0.735 |  | 0.713 | 0.349 |  |  |  |  | 0.728 | 0.579 |  |  |
|  | 34 |  |  | 0.632 | 0.530 | 0.664 |  |  |  | 0.578 | 0.632 |  |  |
|  | 35 | 0.482 |  |  | 0.481 | 0.492 |  |  |  |  | 0.311 |  | 0.281 |
|  | 36 |  |  | 0.767 |  |  |  |  |  | 0.355 |  |  |  |
|  | 37 |  |  | 0.866 | 0.598 | 0.897 |  |  | 0.233 | 0.849 |  |  |  |
| fledge and moult | 38 |  |  |  | 0.431 | 0.720 |  |  |  |  |  |  | 0.362 |
|  | 39 |  |  | 0.658 |  |  |  |  | 0.313 | 0.219 |  |  | 0.373 |
|  | 40 |  |  | 0.067 | 0.106 | 0.549 |  |  | 0.136 | 0.021 |  |  |  |
|  | 41 |  |  | 0.086 | 0.376 | 0.135 |  |  |  | 0.009 |  |  | 0.820 |
|  | 42 |  |  |  |  | <0.001 |  |  | 0.542 |  |  |  |  |
|  | 43 |  |  | 0.008 |  |  |  |  | 0.046 | 0.015 |  |  | 0.521 |
|  | 44 |  |  | 0.002 | 0.157 | 0.149 |  |  | 0.050 | 0.732 |  |  |  |
|  | 45 |  |  | <0.001 | <0.001 | <0.001 |  |  |  | <0.001 |  |  | 0.015 |
|  | 46 |  |  |  |  | 0.743 |  |  | <0.001 |  |  |  |  |
|  | 47 |  |  | 0.011 | 0.004 |  |  |  | 0.010 | 0.017 |  |  |  |
|  | 48 |  |  | 0.471 | 0.001 | 0.252 |  |  |  | 0.089 |  |  | 0.023 |
|  | 49 |  |  |  | <0.001 | 0.002 |  |  | 0.003 |  |  |  |  |
| non-breeding period | 50 |  |  | 0.336 |  |  |  |  | 0.004 | 0.272 |  |  | 0.756 |
|  | 51 |  |  | 0.511 | 0.007 | 0.274 |  |  | 0.072 | 0.069 |  |  |  |
|  | 52 |  |  |  | 0.011 | 0.050 |  |  |  |  |  |  | 0.467 |
|  | 53 |  |  | 0.644 |  | 0.481 |  |  | 0.099 | 0.840 |  |  | 0.763 |
|  | 54 |  |  | 0.194 |  |  |  |  | 0.309 | 0.611 |  |  | 0.531 |
|  | 55 |  |  | 0.088 | 0.005 | 0.792 |  |  | 0.960 | 0.681 |  |  | 0.776 |
|  | 56 |  |  |  | 0.004 | 0.920 |  |  |  |  |  |  |  |
|  | 57 |  |  | 0.887 |  |  |  |  | 0.537 | 0.971 |  |  |  |
|  | 58 |  |  | 0.781 |  | 0.642 |  |  | 0.715 | 0.565 |  |  |  |
|  | 59 |  |  | 0.815 | 0.009 | 0.773 |  |  |  | 0.668 |  |  |  |
|  | 60 |  |  |  |  | 0.471 |  |  | 0.566 |  |  |  |  |
|  | 61 |  |  |  | 0.003 |  |  |  | 0.719 |  |  |  |  |
|  | 62 |  |  |  | <0.001 | 0.969 |  |  | 0.491 |  |  |  |  |
|  | 63 |  |  | 0.801 | 0.006 |  |  |  |  | 0.804 |  |  |  |
|  | 64 |  |  | 0.433 |  |  |  |  |  | 0.823 |  |  |  |
|  | 65 |  |  | 0.616 | 0.004 | 0.587 |  |  |  | 0.840 |  |  |  |
|  | 66 |  |  |  | 0.013 | 0.459 |  |  |  |  |  |  |  |
|  | 67 |  |  | 0.571 |  |  |  |  | 0.615 | 0.991 |  |  |  |
|  | 68 |  |  | 0.798 | 0.019 | 0.496 |  |  | 0.772 | 0.807 |  |  |  |
|  | 69 |  |  | 0.858 | 0.009 | 0.380 |  |  |  | 0.966 |  |  |  |
|  | 70 |  |  |  | 0.031 | 0.275 |  |  | 0.591 |  |  |  |  |
|  | 71 |  |  | 0.855 |  |  |  |  | 0.379 | 0.599 |  |  |  |
|  | 72 |  |  | 0.785 | 0.044 | 0.622 |  |  | 0.447 | 0.734 |  |  |  |
| Bonferroni adjusted P-value | | 0.0022 | NA | 0.0010 | 0.00116 | 0.0011 | NA | NA | 0.0021 | 0.0010 | 0.0023 | NA | 0.0016 |

Table S2: Tukey test results from comparisons between breeders and non-breeder gentoo penguins in each year. Tukey test results conducted when two-way interactions were significant in backward selection procedure for each year and sex in Table 2. Because of multiple comparisons, the P-values were Bonferroni adjusted based on the number of comparisons being conducted. 5-day period 0 covers the period from 1-5 July.

##

## Figure S1

Figure S1: Female Adélie penguin mass changes for breeders and non-breeders throughout each breeding season between 1998/99 and 2002/03 for every 5-day period. Phenology indicated in coloured bars based on range across the five years with annual means indicated by dashed lines for each phenology event. Values represent means ± SEM. Statistical significance from Tukey post-hoc comparisons between breeders and non-breeders for each 5-day period denoted by stars.

**Figure S2**

Figure S2: Male Adélie penguin mass changes for breeders and non-breeders throughout each breeding season between 1998/99 and 2002/03 for every 5-day period. Phenology indicated in coloured bars based on range across the five years with annual means indicated by dashed lines for each phenology event. Values represent means ± SEM. Statistical significance from Tukey post-hoc comparisons between breeders and non-breeders for each 5-day period denoted by stars.

**Figure S3**

Figure S3: Female gentoo penguin mass changes for breeders and non-breeders throughout each breeding season between 2009/10 and 2014/15 for every 5-day period. Phenology indicated in coloured bars based on range across the five years with annual means indicated by dashed lines for each phenology event. Values represent means ± SEM. Statistical significance from Tukey post-hoc comparisons between breeders and non-breeders for each 5-day period denoted by stars.

**Figure S4**

Figure S4: Male gentoo penguin mass changes for breeders and non-breeders throughout each breeding season between 2009/10 and 2014/15 for every 5-day period. Phenology indicated in coloured bars based on range across the five years with annual means indicated by dashed lines for each phenology event. Values represent means ± SEM. Statistical significance from Tukey post-hoc comparisons between breeders and non-breeders for each 5-day period denoted by stars.
